# Omission of Affiliations

**DOI:** 10.1001/jamanetworkopen.2019.1249

**Published:** 2019-03-08

In the Invited Commentary titled “Waiting for Care in Veterans Affairs Health Care Facilities and Elsewhere,”^1^ published January 18, 2019, affiliations were omitted for Drs Fihn and Francis. The Conflicts of Interest Disclosures section should read, “Dr Fihn reports being a former employee of the Department of Veterans Affairs (VA) and continues to perform limited work for the VA under terms of an interagency personnel agreement with the University of Washington. No other disclosures were reported.” The Additional Contributions section should read, “We thank Joe Francis, MD, Department of Veterans Affairs, for thoughtful review. No compensation was received.” This article has been corrected.^1^
